# 

**Published:** 1923-01

**Authors:** 


### HOSPITAL and HEALTH REVIEW

Established 1886 No. 1858. Vol. LXXII.

New Series. Vol. II. No. 16 January 1923 Price Sixpence

CONTENTS.

| | PAGE |
|---|---|
| What We Stand for in Public Health | 65 |
| Notes and Comments—<br>Housing and Unemployment—The Demoralisation of the Dole—The Price of Fog—Smoke Abatement: Germany leads the way—Dirt as an Index of Prosperity—The House Fly—Milk with a Special Label—A Disturbing Milk Circular—Physical Training in Schools—Liverpool's School of Hygiene—The New Dentists' Act—Smallpox Declines—Sterilisation of Tuberculous Sputum—Future of the Poor Law Health Services—Cooperation between a General and a Special Hospital—Seamen's Hospital, Malta, Opens—Leprosy in the United States | 67 |
| The Public Health: Interviews with Local Authorities. — VI. — Portsmouth: Safety First | 73 |
| The Town Planning Acts, 1909 and 1919: Some Difficulties<br>By Edmond R. Abbott | 75 |
| A Visit to Sheffield's Remarkable Sewage Works | 76 |
| Insulin and Diabetes | 78 |
| The Dentists' Act, 1921 | 79 |
| Stopping the Panel Doctor's Pay<br>By R. W. Harris. | 80 |
| National Health Insurance Notes and Comments | 81 |
| The Voluntary Hospitals Commission on Hospital Management | 82 |
| King Edward's Hospital Fund for London | 84 |
| Sir Napier Burnett's Report on the Voluntary Hospitals Outside London | 86 |
| Hospital News | 87 |
| Government Publications | 88 |
| Recent Appointments | 88 |
| Parliamentary News | 89 |
| Reviews | 90 |
| Correspondence | 93 |

